# A Review on Emerging Efficient and Stable Perovskite Solar Cells Based on g-C_3_N_4_ Nanostructures

**DOI:** 10.3390/ma14071679

**Published:** 2021-03-29

**Authors:** Konstantina Gkini, Ioanna Martinaiou, Polycarpos Falaras

**Affiliations:** 1 Institute of Nanoscience and Nanotechnology, NCSR Demokritos, Agia Paraskevi Attikis, 15341 Athens, Greece; k.gkini@inn.demokritos.gr (K.G.); i.martinaiou@inn.demokritos.gr (I.M.); 2 Physics Department, School of Natural Sciences, University of Patras, 26504 Patras, Greece

**Keywords:** perovskite, solar cells, graphitic carbon nitride, carbon based materials, additive engineering, interface engineering, efficiency, stability

## Abstract

Perovskite solar cells (PSCs) have attracted great research interest in the scientific community due to their extraordinary optoelectronic properties and the fact that their power conversion efficiency (PCE) has increased rapidly in recent years, surpassing other 3rd generation photovoltaic (PV) technologies. Graphitic carbon nitride (g-C_3_N_4_) presents exceptional optical and electronic properties and its use was recently expanded in the field of PSCs. The addition of g-C_3_N_4_ in the perovskite absorber and/or the electron transport layer (ETL) resulted in PCEs exceeding 22%, mainly due to defects passivation, improved conductivity and crystallinity as well as low charge carriers’ recombination rate within the device. Significant performance increase, including stability enhancement, was also achieved when g-C_3_N_4_ was applied at the PSC interfaces and the observed improvement was attributed to its wetting (hydrophobic/hydrophilic) nature and the fine tuning of the corresponding interface energetics. The current review summarizes the main innovations for the incorporation of graphitic carbon nitride in PSCs and highlights the significance and perspectives of the g-C_3_N_4_ approach for emerging highly efficient and robust PV devices.

## 1. Introduction

In today’s industrial societies, energy needs are constantly growing, and become harder to fulfill. Energy production comes mostly from the combustion of fossil fuels, and has a major impact on climate change [1]. Solar energy is one of the most significant sources of renewable energy and researchers have extensively endeavored to use it in order to address these concerns [2]. Solar energy is characterized by abundance and ease of use, and have already showed a great potential to be a prime source of energy in the future [3]. In recent years, it has attracted scientific and technological interest in the direction of its utilization and efficient conversion into other useful forms of energy. Photovoltaic (PV) devices are the basic technology for this process. The technologically mature PV panels based on silicon (Si) already occupy a large share in global energy production/consumption. Nevertheless, the implementation of the Si-based technology is limited by high fabrication cost of the corresponding modules and panels [4]. Additionally, the commercialization of the 2nd generation solar cells (Si, CdTe, CIGS) is, mainly, hindered due to module stability issues [5].

**Figure 1:**
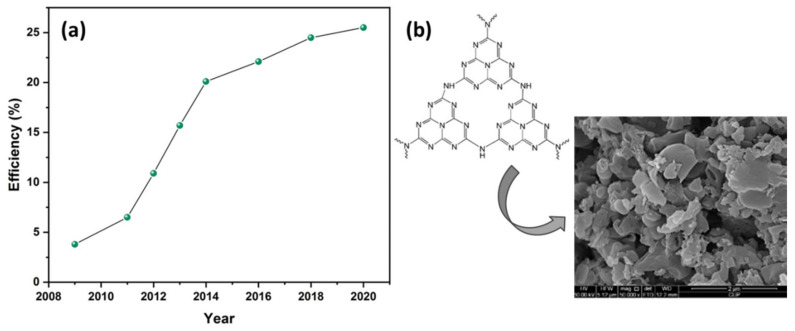
(**a**) Perovskite solar cells’ (PSCs’) certified efficiency evolution 2009–2020 and (**b**) the structure and morphology (SEM image) of graphitic carbon nitride (g-C_3_N_4_). Reproduced with permission from [27], Copyright 2014, Elsevier.

Recent developments in photovoltaic research have shown that silicon cells, which still dominate the market, can be replaced by new cells based on more efficient and cheaper materials, such as perovskites. Lately, the scientific community has focused on this type of 3rd generation cells, as their near-ideal optoelectronic properties [6], including the tunable band gap, high carrier mobility [7], long carrier lifetime [8], and the solution-processed and low-cost fabrication methods [9], make them very promising as light absorbers. The power conversion efficiency (PCE) of perovskite solar cells (PSCs) has increased rapidly in recent years, exceeding 25% within only a few years of development [10,11,12,13,14,15,16] (Figure 1a), surpassing other 3rd generation PV technologies. However, several issues including further efficiency increase and performance stabilization have not been effectively addressed yet. In fact, grain size and crystallinity of the perovskite films, conductivity of the functional layers, charge recombination, surface and intrinsic defects, hysteresis phenomena, and chemical/thermal stability under continuous sun illumination in atmospheric environment, are main issues that require to be effectively addressed. Research interventions aiming for further development of these photovoltaic devices have in common the integration of innovative nanostructured materials in order to increase their PCE, improve their long-term stability and decrease their fabrication cost. Among this purpose, several strategies, such as materials and interface engineering [17,18,19,20,21,22,23,24,25,26], have been reported.

Recently, graphitic carbon nitride (g-C_3_N_4_) (Figure 1b) [27] has been the prime center for consideration in the development of PSCs, due to its thermal and chemical stability, its trap-healing ability and its high corrosion resistance [28]. Additionally, g-C_3_N_4_ is an n-type semiconductor with narrow bandgap (≈2.7 eV), and is characterized by easy and low cost fabrication and nontoxicity [29]. Its outstanding physical and chemical properties and its ability to be formed in several nanostructures (nanosheets, nanotubes, etc.) [30,31] are the reason that g-C_3_N_4_ has been widely used in various applications, such as pollutant degradation [32], CO_2_ reduction [33,34], water splitting [35,36], photocatalytic applications [31,37,38,39,40], capacitors [41,42], and solar cells [43,44,45,46,47,48]. In particular, integration of graphitic carbon nitride into PSCs has drawn special attention, providing devices with potential low fabrication cost, high chemical stability, and feasibility of structure modification via surface engineering [49,50]. The highest reported efficiency of g-C_3_N_4_ [51] based PSCs is over 22%, indicating that the incorporation of g-C_3_N_4_ in PSCs is a very promising approach that needs further investigation and optimization. Since the first report of g-C_3_N_4_ as photocatalyst for visible-light photocatalytic water-splitting in 2009 by Wang et al. [52] g-C_3_N_4_ has been commonly used as photocatalyst, however, Xu et al. prepared high quality g-C_3_N_4_ layered films by thermal condensation of a liquid supramolecular precursor and confirmed their successful application as acceptors in P3HT-based organic solar cells with V_oc_ values higher than 1V [53]. The use of g-C_3_N_4_ in organic photovoltaics (OPVs) generally leads to high V_oc_ values as a result of Fermi level adjustment and effective charge transfer [43,45,53]; however, the thickness of the g-C_3_N_4_ layer might affect the device performance [53] and needs further optimization. With the utilization of ultrathin g-C_3_N_4_ films the PCE was improved by 70% [45]. In addition, g-C_3_N_4_ has been employed in CdS quantum dot sensitized solar devices [44] and in dye-sensitized solar cells (DSSCs) [54] enhancing significantly the PCE due to minimized reverse tunneling probability and delayed backward transportation of electrons between TiO_2_ and dyes.

In this review, we present an overview of the successful integration of graphitic carbon nitride in PSCs which led to improved charge transport, reduced defects, suppressed carrier recombination, and facilitated the perovskite crystal growth, either as additive in perovskite materials and electron transport materials (ETLs) or as modification at the interfaces ETL/perovskite and perovskite/HTL (hole transport layer) [55,56].

## 2. Working Principles and Challenges of PSCs

**Figure 2:**
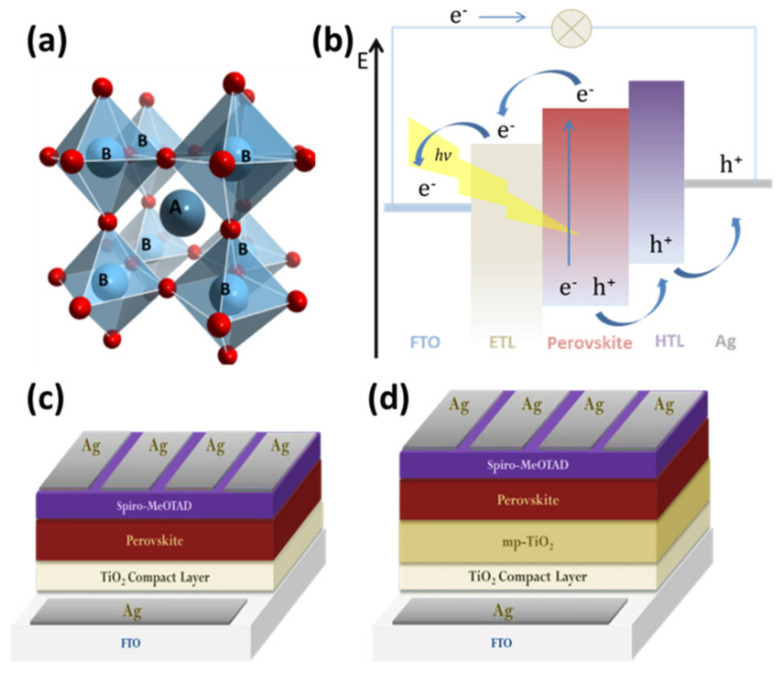
(**a**) Illustration of the general perovskite crystal structure, (**b**) the working mechanism of a PSC, and (**c**,**d**) the two typical device architectures; planar and mesoporous, respectively. Reproduced with permission from [73], Copyright 2019, Elsevier.

Perovskite materials have been studied either as sunlight absorbers [57] or as hole transport materials [58] in photovoltaic cells, due to their special properties, such as the high absorption coefficient [7,59], the high mobility load carriers [60], long charge carrier diffusion length [61], wide absorption spectrum, and configurable energy gap. Perovskite materials exhibit the stoichiometry ABX_3_ [62]. For photovoltaic applications, A is usually an organic cation, such as methylammonium (CH_3_NH_3_^+^) [63] and formamidinium (HC(NH_2_)^2+^) [64], or an inorganic, such as Cs^+^, or a combination of them, B is a metal cation (usually Pb^2+^ or Sn^2+^), while the X position represents a non-metallic anion such as the halogens Cl^−^, Br^−^, I^−^ and the combination of these [65]. Figure 2a [66] illustrates the crystal structure of ABX_3_.

The PSC’s working mechanism includes the absorption of photons [67] from the absorber/perovskite and the transfer and extraction of charge carriers to the respective electrodes. Specifically, photons’ absorption is followed by the presence of excited electrons in the conduction band and holes in the valence band of the perovskite. The electrons are injected into ETL and migrate to the anode. Subsequently, the electron passes through the external circuit to the cathode. At the same time, the holes are transferred through the hole transport layer (HTL) and migrate to the cathode, where electrons and holes recombine (Figure 2b). Charge carrier extraction occurs at the perovskite/ETL and perovskite/HTL interfaces and, then, at the ETL/anode and HTL/cathode interfaces. Thus, the overall photovoltaic performance of PSCs is greatly affected by the properties of these interfaces. Two types of PSC device architectures exist, depending on the ETL and HTL ordering. The case of the ETL, which is an n-type semiconductor, being placed before the perovskite and the HTL, which is a p-type semiconductor, after perovskite attributes to the n–i–p structure, while the opposite attributes to the p–i–n structure [68,69,70]. In the case of the n-i-p type PSCs, planar [71,72] or mesoporous [14,73] configurations can be distinguished, depending on the morphology of the ETL material (Figure 2c,d). Nevertheless, in addition to the perovskite absorber deposited on the ETL, a complete PSC device also comprises the electron collection substrate, usually a conducting oxide glass such as fluorine-doped tin oxide (FTO) or indium tin oxide (ITO) and the hole transporting layer (HTL) in contact with a metallic (Au, Ag) or carbon upper electrode [74,75].

Nowadays, PSCs have already reached a high efficiency in a very short period of research activity; however, there are other factors that also need to be addressed. First of all, during the charge carriers’ transfer and extraction through the successive layers, excess of carriers are not collected and recombine (radiatively or nonradiatively) at the interfaces [76,77]. Other challenges in the field of PSCs are the thermal and chemical stability [78] of the constituent materials and the overall device, hysteresis phenomenon [79,80], intrinsic and surface defects [81,82], enhanced charge carriers’ mobility and lead’s toxicity. Towards the mitigation of these problems a number of material and interface engineering approaches have been proposed including the integration of reduced graphene oxide as additive in the ETL, the perovskite and the HTL, the passivation of the ETL’s surface with metallated porphyrins and organic dyes, the modification of the titania ETL with transition metals, such copper and niobium, the passivation of perovskite layer’s surface with formamidinium iodide solution in isopropyl alcohol, and the addition of 4-tert-butylpyridine (tBP) in perovskite precursor as surface modification agent [17,18,19,20,21,22,23,24,25,26]. The incorporation of graphitic carbon nitride has been proved to be an efficient method for controlling crystal growth, passivating defects and reducing charge carriers’ recombination rate, and, thus, confronting many of the above-mentioned challenges.

## 3. Properties of g-C_3_N_4_

Intense theoretical and experimental interest has been focused on materials with tunable properties. Among these, carbon nitrides have attracted great attention due to their relative stability, structure and physicochemical properties [83]. In particular graphitic carbon nitride (g-C_3_N_4_) as a pyrolysis product of nitrogen-rich precursors, such as dicyandiamide, melamine, thiourea, and urea has attracted significant interest among the research community.

**Figure 3:**
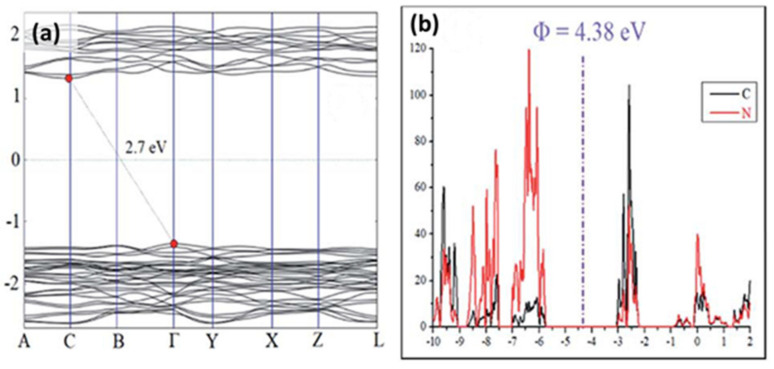
(**a**) g-C_3_N_4_ band structure from density of states (DOS) calculations and (**b**) analysis of partial density of states (PDOS) comprising C and N orbitals. Reproduced with permission from [42], Copyright 2018, Elsevier.

Graphitic carbon nitride presents very interesting structural and oproelectronic properties. Following polymerization, the existing basic core triazine C_3_N_3_ (melam) and tri-s-triazine C_6_N_7_ (melem) planar units create a layered structure similar to graphite. The typical XRD peaks are placed at 2θ ~27° and 2θ ~13° [84], from which the first is related to interlayer stacking of the (002) melem planes and the second is attributed to the (100) planes corresponding to inplane ordering of the nitrogen-linked heptazine units. As in the case of graphite, the C_3_N_3_ and C_6_N_7_ cores comprise six-membered rings with sp^2^ C-N hybridization presenting intrinsic structural vacancies surrounded by nitrogen atoms [49]. Depending on the precursor and the conditions of the pyrolysis used, the *g*-C_3_N_4_ powders have the possibility to result in different structures from highly crystalline samples to bulk (high temperature) and tubular (low temperature) samples [85,86]. In fact, DFT calculations were performed on carbon nitride single-layer to evaluate the structural and optoelectronic properties. The obtained results are consistent with a layered structure of slightly buckled heptazine cores, where the observed distortion can be attributed to electrostatic repulsions on the triazine-rings involving the two-coordinated N atoms with lone pair electrons [87]. Density of states (DOS) calculations (Figure 3a) have demonstrated that g-C_3_N_4_ is a semiconducting material having a moderate band gap of 2.7 eV. Moreover, the corresponding analysis of partial density of states indicates the position of the valence band and the conduction band at −5.74 and −3.07 eV (vs. vacuum), respectively (Figure 3b) [35,42].

So far, numerous methods have been used in order to modify the surface of bulk graphitic carbon nitride for different purposes. Chemical and thermal exfoliation [88] has been used in order to increase the active surface area as well as the optical properties. It becomes apparent that the exfoliation of bulk graphitic carbon nitride into 2D structure yields in an elevated bandgap, in agreement with the observed color modification of the samples to light yellow (from dark ones). [89,90]. Doping with heteroatoms [91] leads to an enhancement of the electrical conductivity and photoactivity due to the substitution of C or N by B, S, and Fe atoms of different valence in the honeycomb lattice. Such substitution introduces supplementary states in the density of states and usually involves nitrogen and boron as doping elements as their valence number is closer, in comparison to carbon atoms [92]. Finally, the introduction of functional groups leads to improved electrochemical properties as a result of changes in conductivity resulting from delocalized π-electrons, adsorbed species and defect sites [93]. Thus, the research community has focused on graphitic carbon nitride (g-C_3_N_4_) as a metal free, nontoxic and VLA (visible light activated) photocatalyst with a unique 2D structure, outstanding chemical stability and tunable physicochemical properties [55,56]. Compared to other materials reported in literature for PSCs engineering, the advantages of g-C_3_N_4_ can be attributed to its tunable structural and optoelectronic properties which can be optimized to the needs of the PSC technology. The carbon nitride material, characterized by easy and low-cost fabrication, also disposes high chemical and thermal stability that is required for the long-term operation of robust PSC devices. In general, the incorporation of bulk or modified g-C_3_N_4_ into solar cells is desirable in order to achieve highly stable and efficient performances.

## 4. Incorporation of g-C_3_N_4_ in PSCs

Recent studies have shown that nitrogen-rich precursors demonstrated enhanced PCE for PSCs. It seems that the incorporation of nitrogen functionalities has resulted in effective passivation of organic–inorganic halide perovskite materials [55,56]. In 2017, Lee et al. [94] reported that the defect sites of methylammonium lead tribromide (MAPbBr_3_) were passivated through coordinate bonding between the nitrogen atoms and undercoordinated lead ions, by using amine-based treatment on top of the perovskite film. In another study by Hsieh et al. [55], they used urea and thiourea, previously dissolved in DMF at a concentration of 25 wt% and stirred overnight, as additives in MAPbI_3−*x*_Cl*_x_* and MAPbI_3_ perovskite solar cells. In both cases, the PCE was improved and the MAPbI_3_ based PSC presented a PCE of 18.8% (from 14.6% in the reference cell). The authors characterized the additives containing perovskite absorbers using UV-vis spectroscopy, scanning electron microscopy and grazing incidence wide angle X-ray diffraction measurements and attributed the observed performance enhancement to reduced grain boundaries of MAPbI_3_ hindering charge recombination and allowing the effective charge carriers (electrons and holes) transport. Similarly, by incorporating 4mol% of urea (a bifunctional nonvolatile Lewis base) in MAPbI_3_, Lee et al. [95] obtained significantly enhanced photoluminescence lifetime accompanied by suppression of the trap-related nonradiative recombination. XRD analysis and FTIR spectroscopy were used to monitor both the perovskite growth and intramolecular interactions. In parallel, time-resolved photoluminescence (TRPL) measurements permitted the determination of charge-carrier lifetime and trap densities. Thus, the interaction of urea with the perovskite precursors in solution was confirmed. The urea additive retards growth and enhances perovskite crystallinity, and then passivates defects in the absorber by precipitating at the grain boundaries.

**Figure 4:**
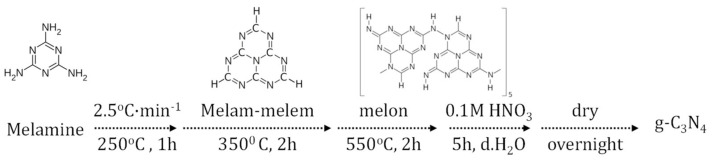
Schematic diagram of a typical pyrolysis procedure for g-C_3_N_4_ fabrication.

Graphitic carbon nitride is a pyrolysis product of nitrogen-rich precursors (Figure 4). Its incorporation in PSCs has attracted scientific attention only in the few last years. However, the up-to-now findings appear to be promising and of high interest for the emerging perovskite solar cells.

### 4.1. g-C_3_N_4_ Modified Perovskite Absorbers

**Figure 5:**
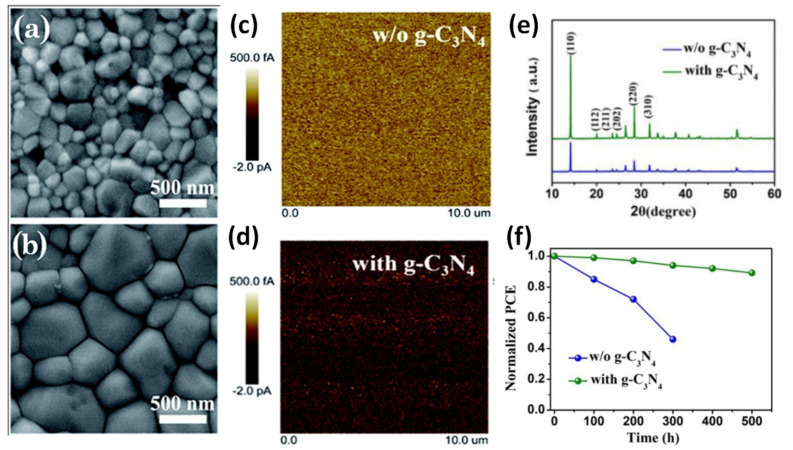
Top-view SEM images of the perovskite films based on (**a**) control MAPbI_3_ and (**b**) 0.1 wt% g-C_3_N_4_ modified MAPbI_3_ film, conductive AFM images of (**c**) control MAPbI_3_ and (**d**) 0.1 wt% g-C_3_N_4_ modified MAPbI_3_ films with a scanning area of 10 × 10 μm^2^, (**e**) XRD patterns of control and 0.1 wt% g-C_3_N_4_ modified MAPbI_3_ films, and (**f**) operational stability of the encapsulated PSCs fabricated with or without g-C_3_N_4_ under constant 1 sun illumination. Reproduced with permission from [96], Copyright 2019, The Royal Society of Chemistry.

In the few past years many studies have focused on the incorporation of g-C_3_N_4_ into the perovskite layer as a passivation strategy for the improvement of the crystallinity, which is a complicated process, the conductivity and, thus, the overall performance of the devices. Towards this strategy, the work from Liao et al. [96] refers to the incorporation of a small amount of g-C_3_N_4_ derived by standard pyrolysis of dicyandiamide, into the perovskite ink, which revealed impressive PCEs up to 21.1%. The addition of g-C_3_N_4_ led to compact perovskite films with passivated defects, large grain sizes, improved conductivity, and high crystallinity (Figure 5a–e), as confirmed by SEM, FTIR, XPS, and c-AFM measurements, which led to devices with considerably low charge recombination rate. The g-C_3_N_4_ modified PSCs are characterized by extremely low hysteresis accompanied by enhanced stability. In fact, after 500 h continuous illumination under one sun, only about 10% decline in devices PCE was observed (Figure 5f).

**Figure 6:**
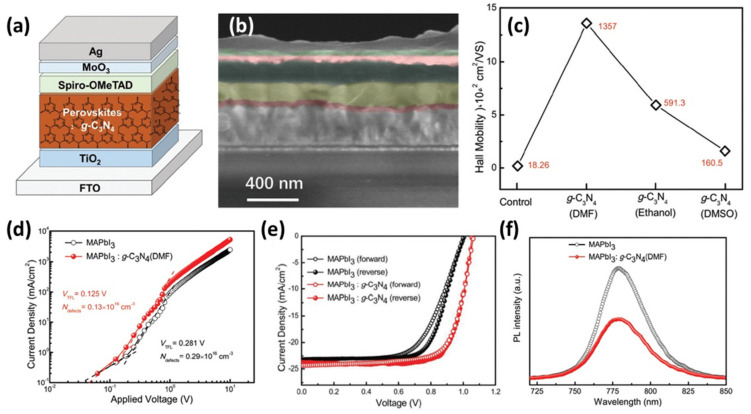
(**a**) Architecture of MAPbI_3_:g-C_3_N_4_ based n-i-p PSC and (**b**) corresponding cross-sectional SEM image, (**c**) Hall mobility of MAPbI_3_ and MAPbI_3_:g-C_3_N_4_ in various solvents films, (**d**) current density–voltage (J–V) curves of hole-dominated devices with a structure of FTO/PEDOT:PSS/MAPbI_3_ (with and without g-C_3_N_4_)/MoO_3_/Ag, (**e**) J–V curves of MAPbI_3_ and MAPbI_3_:g-C_3_N_4_ based devices by forward and reverse scan, and (**f**) photoluminescence (PL) spectra of MAPbI_3_ and MAPbI_3_:g-C_3_N_4_ (DMF) films deposited on TiO_2_/FTO. Reproduced with permission from [56], Copyright 2017, Wiley-VCH GmbH.

**Figure 7:**
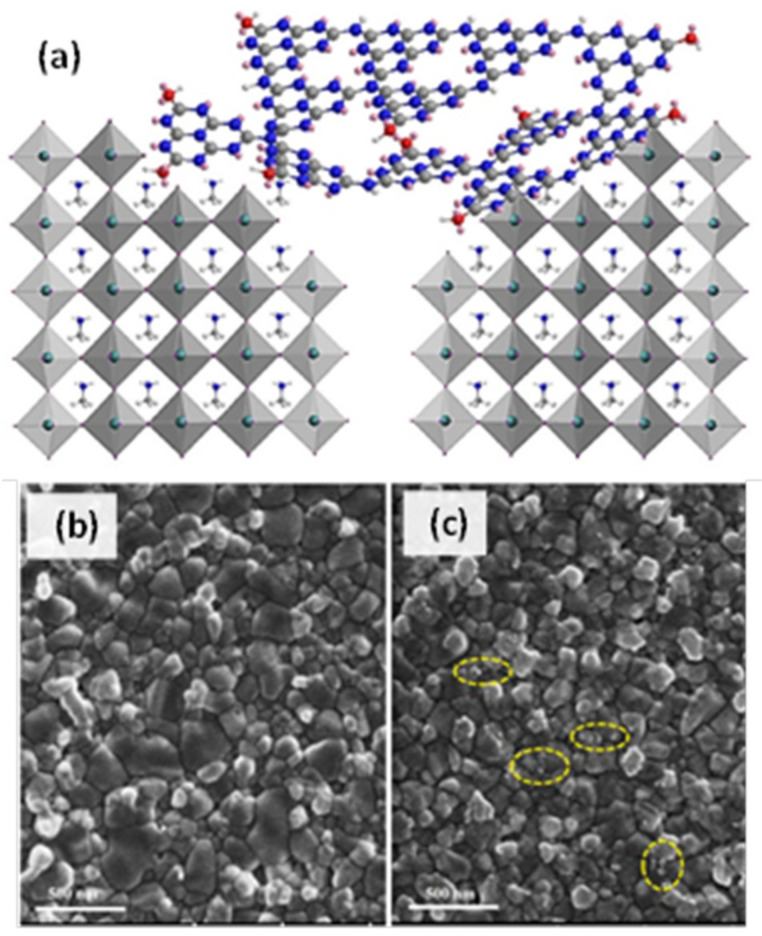
(**a**) Schematic illustration of the self-location process of E-g-C_3_N_4_ at MAPbI_3_ grain boundaries by hydrogen bonding interaction. (**b**,**c**) Top-view FESEM images of MAPbI_3_ film and E-g-C_3_N_4_ modified MAPbI_3_ film, respectively. Reproduced with permission from [97], Copyright 2019, Elsevier.

Similarly, in a previous work Jiang et al. [56] reported that the addition of 0.6 wt% pure sheets of g-C_3_N_4_ produced by standard pyrolysis of urea into MAPbI_3_ layer (Figure 6a,b) increased the crystallization and grain sizes of the perovskite film, proved by SEM characterization, as well as, its carrier mobility (Figure 6c). Thus, conductive AFM measurements confirmed the improvement of the corresponding perovskite absorber/spiro HTM interface. In parallel, the defects density was reduced ((Figure 6d) which consequently reduced hysteresis (Figure 6e) and increased the PCE from 16.22% to 19.49%. In this work the authors examined thoroughly the impact of different solvents, which were used to dissolve g-C_3_N_4_, on the perovskite’s crystal growth and managed to control the crystallization and reduce the charge recombination (Figure 6f). Among the employed solvents (DMF, DMSO, ethanol), DMF did not show to significantly affect the cell parameters. In a similar vein, Wei et al. [97] prepared ultrafine E-g-C_3_N_4_ nanoparticles, using melamine precursor and annealing at 550 °C followed by intercalation in H_2_SO_4_ and fast stripping in NH_3_ aqueous solution. The obtained nanomaterials, self-located at the MAPbI_3_ grain boundaries via hydrogen bonding interaction (Figure 7), worked as prohibitory of electron-hole recombination. Thus, they were successfully incorporated into PSCs improving the device performance by 35%.

The presence of integrated graphite carbon nitride is shown by FESEM top-view images (Figure 7b,c) of the modified MAPbI_3_ films demonstrating that most of the ultrafine E-g-C_3_N_4_ nanoparticles are located on the grain boundaries. During the crystallization process often a large number of electron trap sites is created, leading to electron loss and charge recombination. The ultrafine exfoliated graphitic carbon nitride (E-g-C_3_N_4_) nanoparticles are covered with N-H or O-H groups and, thus, they are easily adsorbed by forming hydrogen bonds with N-H bonds present on the perovskite grain boundaries. These nanoparticles were applied as intermediate materials between the perovskite absorber and the HTM) and resulted in increased PCE by passivizing the defects at grain boundaries.

Yang et al. in 2019 [98] implemented g-C_3_N_4_ into carbon-based PSCs and reported that the addition of g-C_3_N_4_ into the precursor solution of the perovskite can result in improved surface morphology and larger grain size, and, thus, obtain high crystal quality of perovskite film. Specifically, the addition of 0.5wt% g-C_3_N_4_ reduced the root-mean-square roughness (RMS) from 15.3 nm, for the pristine perovskite, to 11.5 nm, and increased the average grain size from 150 nm, for the pristine one, to 270 nm determined by SEM and AFM measurements. The PCE of carbon based PSCs was significantly improved from 10.5% to 12.8% as a result of crystal quality improvement. In addition to this, in order to reduce the charge recombination, they spin-coated an Al_2_O_3_ insulating layer on the ETL surface. This modification at the interface between ETL and perovskite reduced the charge carrier recombination and resulted in an increased PCE of 14.3%.

**Figure 8:**
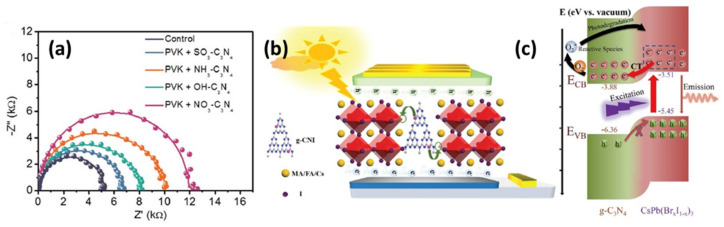
(**a**) Nyquist plot of control and devices passivated by functionalized C_3_N_4_. Reproduced with permission from [99], Copyright 2019, Wiley-VCH GmbH. (**b**) Schematic diagram of the mechanism of g-CNI modified PSCs. Reproduced with permission from [100], Copyright 2019, The Royal Society of Chemistry. (**c**) Schematic illustration of charge transfer between CsPb(Br_x_I_1−x_)_3_ nanocrystals and g-C_3_N_4_. Reproduced with permission from [101], Copyright 2020, Wiley-VCH GmbH.

Li et al. in 2019 [99] employed various passivation agents as perovskite additives including sulfonic, amino, nitrato, and hydroxy organic groups incorporated in g-C_3_N_4,_ prepared using melamine as precursor following a heat treatment process and further cure in sulfuric acid. The authors investigated the materials morphology with top-view SEM, their optoelectronic properties with steady state (PL), time-resolved (TRPL) photoluminescence and UV–vis absorption spectra, as well as the photovoltaic performance of the resulting PSCs with current density–voltage (J–V) curves. Improved nucleation and crystallinity were observed following successful g-C_3_N_4_ functionalization of the perovskite expressed by grain size increase, energy disorder decrease and efficient passivation of the absorber. The devices based on perovskites functionalized by graphitic carbon nitride present PCE values as high as 20.08%, clearly outperforming those without passivation. This was understood in terms of greater charge mobility and was further supported by Nyquist plots (Figure 8a) confirming higher values of charge recombination resistance. Furthermore, the doping of iodine in graphite carbon nitride and its incorporation to the triple cation perovskite film was investigated by Cao et al. [100], who synthesized g-CNI by direct mixing dicyandiamide with ammonium iodide and subsequent heat treatment at 550 °C. As a result, they obtained perovskite films with high crystallinity and low trap states density. Improved photovoltaic performance of the corresponding PSCs was reported, attributed to the interaction of the iodide of the g-CNI group with the under-coordinated Pb ions on both the surface and at the grain boundaries of perovskite absorber (Figure 8b). Following optimization of the g-CNI concentration, the modified devices showed PCE as high as 18.28%, significantly higher than that of the reference cell. Based on XRD measurements showing that the lattice diffraction peaks (in shape and position) of the two perovskites (modified and reference) are very similar, the authors suggested that g-CNI was not inserted in the perovskite lattice but is located on the material surface or at the grain boundaries.

In order to further study g-C_3_N_4_ and its effect on energy/charge transfer, Sheng et al. [101] prepared heterostructures of CsPb(Br_x_I_1−x_)_3_ and g-C_3_N_4_ nanosheets with the standard procedure from melamine and reported an enhancement of charge separation and transportation due to special band alignments (Figure 8c) revealed by stead-state photoluminescence (PL) spectra, time-resolved PL spectra, and photodegradation- and temperature-dependent PL results. Compared to other heterostructures, the heterostructures formed by perovskite nanocrystals (PNCs) and g-C_3_N_4_ nanosheets (CN) not only broaden the absorption spectra, but also enhance the absorption capacity. The efficient electron–hole separation by charge transfer lead to a significant charge transfer efficiency up to 98.16%. In general, the utilization of g-C_3_N_4_ as additive or in combination with functionalized groups as a passivation strategy improves significantly the overall photovoltaic performance by suppressing charge recombination.

### 4.2. g-C_3_N_4_ as Charge Carrier Transport Material

The most significant research efforts have focused on the addition of g-C_3_N_4_ into the perovskite absorber, however, lately a few studies came out using it as electron transport layer (ETL) or/and hole transport layer (HTL). As the number of investigations dealing with the application of g-C_3_N_4_ in the ETL of PSCs increases, more promising results are expected by this emerging, innovative and most of all promising technological approach. In 2019 for the first time, to the author’s knowledge, Chen et al. [51] developed a hybrid ETL nanocomposite consisting of SnO_2_ with g-C_3_N_4_ quantum dots (QDs). The synthesis includes treatment with H_2_SO_4_ and HNO_3_ acids as well hydrothermal cure in autoclave with NH_3_·H_2_O in order to form porous graphitic carbon nitride quantum dots (g-CNQDs). In this way multiple benefits were achieved. Firstly, the intrinsic interface crystal defects of SnO_2_ were reduced.

**Figure 9:**
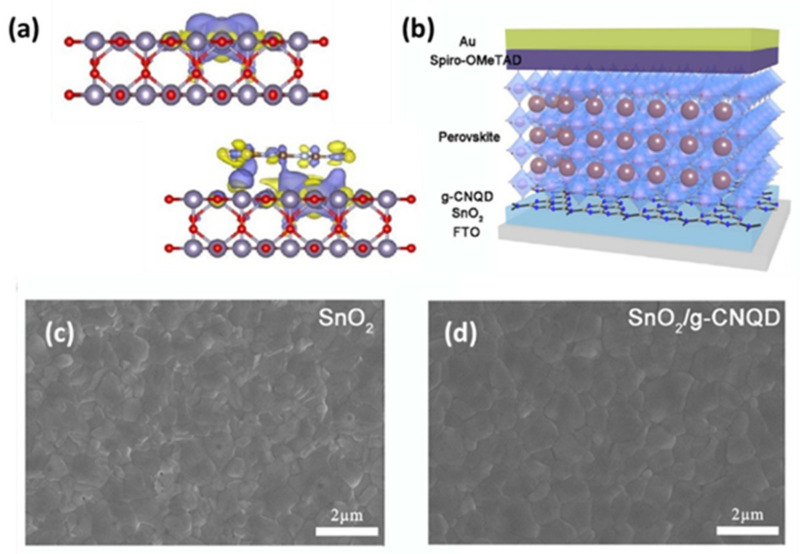
(**a**) The side view for the charge density difference of SnO_2_ (up) and G-SnO_2_ (down) with oxygen vacancy, reproduced with permission from [51], Copyright 2020, Elsevier. (**b**) Schematic illustration of the PSCs with g-CNQD modified SnO_2_ layers, and top-view SEM images of perovskite films based on (**c**) SnO_2_ and (**d**) SnO_2_/g-CNQD ETL. Reproduced with permission from [102], Copyright 2020, The Royal Society of Chemistry.

In fact, the lone-pair electrons of nitrogen can interact with the under-coordinated Sn to effectively passivate the defects of SnO_2_ related to oxygen-vacancies (Figure 9a). This resulted in a PCE exceeding 22%, which was attributed to the ability of g-C_3_N_4_ for trap-healing, its high resistance to corrosion and its significant chemical stability. In addition, devices based on the G-SnO_2_ electron transport layer show exceptional long-term stability, retaining 90% of their initial performance when they are stored for 1500 h in a humid environment (with about 60% humidity). In conclusion, the utilization of g-C_3_N_4_ as ETL in perovskite solar cells enhances significantly both the performance and the long-term stability. In a following study, Liu et al. [102] also used graphitic carbon nitride quantum dots (g-CNQDs) as modifying reagent at the SnO_2_/perovskite interface (Figure 9b). The g-CNQDs were synthesized by a heat treatment of urea and sodium citrate. This led to the decrease of the SnO_2_ surface roughness, less grain boundaries and the facilitation of the crystal growth of perovskite absorber (Figure 9c,d) as detected by SEM measurements. The best PSC exhibited a PCE of 21.23% with negligible hysteresis. Furthermore, the long-term stability of the device was evaluated after being stored in ambient air under room light with a humidity of ≈30% upon 30 days’ exposure. Impressively, the PCE of g-CNQD modified PSC kept over 90% of initial performance after 30 days. Another group innovated by modifying the interfaces of ETL/perovskite and perovskite/HTL with the addition of g-C_3_N_4_ derived from urea. Liu et al. [103] addressed that the g-C_3_N_4_ modification does not exhibit desirable band alignments among ETL, HTL, and MAPbI_3_ perovskite absorber at the two interfaces; the authors observed an outstanding PCE increase of 19.69% for the dual incorporated PSC over the 18.03% of the reference device. The performance improvement was assigned to the reduction of the trap density proved by TEM and SEM evaluation at both ETL/perovskite and perovskite/HTL interfaces. Overall, the addition of g-C_3_N_4_ either as additive in the perovskite and/or as a modification at the ETL/HTM transport layer leads to higher efficiencies (Table 1) and long-term stability of the perovskite solar cells.

## 5. Outlook and Conclusions

In the last decade, the amount of research studies dealing with cost-effective perovskite solar cells has increased tremendously. The efficiencies of these devices have exceeded 25% in a short time. However, there are, still, challenges associated with charge carrier recombination both in the perovskite and at the interfaces within the device, and with long-term PSC device stability. In this way, researchers incorporated graphitic carbon nitride in PSCs due to its suitable properties. Graphitic carbon nitride (g-C_3_N_4_) is very promising for application in devices operating under direct sun illumination, as the material has an energy gap (Eg) in the visible domain. In addition, the existing nitrogen sites seem to enhance crystallinity, reduce grain boundaries and result in defects passivation and charge carrier transport facilitation. Besides, g-C_3_N_4_ is highly chemically and thermally stable as a material, a fact that renders this material favorable for PSCs’ applications.

This approach emerged only a few years before and the most promising works deal with the use of g-C_3_N_4_ as an additive or modification at the perovskite materials and layers, respectively. Lately, very few studies have been conducted on the impact of g-C_3_N_4_ as an alteration of the ETL and the HTL. In spite of the impressive enhancement on efficiency and stability, significant scientific problems and technological challenges still remain, requiring complete answers and adequate solutions, such as the controllable thin film growth and deposition, the scalable fabrication process and results reproducibility, lead toxicity and the current–voltage hysteresis as well as degradation under illumination. The addition of g-C_3_N_4_ in perovskite solar cells improved significantly the PCE of the devices in all cases and this is attributed to the main following facts. Charge recombination was restrained, the crystallization and the grain size of the perovskites increased and the conductivity at the interfaces was enhanced. Thus, g-C_3_N_4_ could be referred to as a universal material for overall optimization of the PSCs. Although g-C_3_N_4_ has been comprehensively studied in the field of photocatalysis, the optoelectronic devices based on g-C_3_N_4_ are still in their infancy. We advocate that further research on better controlling the g-C_3_N_4_ nanostructures’ fabrication (optoelectronic properties) and the deposition method at the interfaces (wetting behavior) could lead to novel PSC devices with high power conversion efficiency and increased stability. Thus, the multifunctional character of graphitic carbon nitride and its use as a universal material in PSCs is expected to overcome limitations and address challenges usually encountered with relevant approaches employing carbon nanostructures, dyes, transition metals, and/or solution additives.

## Figures and Tables

**Table 1:** Recent development of g-C3N4 based PSC photovoltaic performance.

Structure	Jsc (mA∙cm^−2^)	Voc (V)	FF (%)	PCE (%)	Ref.	Year
FTO/compact TiO_2_/g-C_3_N_4_ modified MAPbI_3_/spiro-OMeTAD/MoO_3_/Ag	24.31	1.07	74.0	19.49	[56]	2018
FTO/compact TiO_2_/g-C_3_N_4_ modified MAPbI_3_/spiro-MeOTAD/Au	23.00	1.16	79.0	21.10	[96]	2019
FTO/compact TiO_2_/exfoliated g-C_3_N_4_ modified MAPbI_3_/spiro-OMeTAD/Au	23.20	1.10	62.0	15.80	[97]	2019
FTO/c-TiO_2_/m-TiO_2_/Al_2_O_3_/MAPbI_3_ + 0.5 wt% g-C_3_N_4_/carbon	23.80	1.00	60.1	14.34	[98]	2019
FTO/c-TiO_2_/m-TiO_2_/MAPbI_3_ + 0.5 wt% g-C_3_N_4_/carbon	24.00	0.92	58.2	12.85		
FTO/TiO_2_/g-C_3_N_4_ modified CsFAMAPbI_3−x_Br_x_/spiro-OMeTAD/Au	22.63	1.06	73.0	17.53	[100]	2019
FTO/TiO_2_/iodine doped g-C_3_N_4_ modified CsFAMAPbI_3−x_Br_x_/spiro-OMeTAD/Au	22.97	1.07	74.0	18.28		
ITO/PTAA/CsFAMAPbI_3−x_Br_x_ + NO_3_ functionalized g-C_3_N_4_/PCBM/BCP/Ag	22.84	1.11	79.20	20.08	[99]	2019
ITO/PTAA/CsFAMAPbI_3−x_Br_x_ + SO_3_ functionalized g-C_3_N_4_/PCBM/BCP/Ag	22.47	1.06	75.96	18.09		
ITO/PTAA/CsFAMAPbI_3−x_Br_x_ + NH_3_ functionalized g-C_3_N_4_/PCBM/BCP/Ag	22.56	1.07	76.96	18.58		
ITO/PTAA/CsFAMAPbI_3−x_Br_x_ + OH functionalized g-C_3_N_4_/PCBM/BCP/Ag	22.37	1.08	75.68	18.28		
ITO/g-C_3_N_4_ QDs modified SnO_2_/CsFAMAPbI_3−x_Br_x_/Spiro-MeOTAD/Au	24.03	1.18	78.3	22.13	[51]	2020
FTO/SnO_2_/g-C_3_N_4_/MAPbI_3_/g-C_3_N_4_/Spiro-OMeTAD/Au	21.45	1.14	80.7	19.67	[103]	2020
FTO/SnO_2_/g-C_3_N_4_ QDs/(FA/MA/Cs)PbI_(3−(x+Y))_Br_(x)_Cl_(y)_/Spiro-OMeTAD/Au	23.39	1.14	79.6	21.23	[102]	2020

## Data Availability

Data sharing not applicable.
